# Misleading Westerns: Common Quantification Mistakes in Western Blot Densitometry and Proposed Corrective Measures

**DOI:** 10.1155/2019/5214821

**Published:** 2019-01-21

**Authors:** Trent A. J. Butler, Jonathan W. Paul, Eng-Cheng Chan, Roger Smith, Jorge M. Tolosa

**Affiliations:** ^1^Mothers and Babies Research Centre, School of Medicine and Public Health, Faculty of Health and Medicine, University of Newcastle, Callaghan, NSW, 2308, Australia; ^2^Hunter Medical Research Institute, 1 Kookaburra Circuit, New Lambton Heights, NSW, 2305, Australia; ^3^John Hunter Hospital, New Lambton Heights, NSW, 2305, Australia

## Abstract

Densitometry data generated for Western blots are commonly used to compare protein abundance between samples. In the last decade, it has become apparent that assumptions underpinning these comparisons are often violated in studies reporting Western blot data in the literature. These violations can lead to erroneous interpretations of data and may contribute to poor reproducibility of research. We assessed the reliability of Western blot data obtained to study human myometrial tissue proteins. We ran dilution series of protein lysates to explore the linearity of densitometry data. Proteins analysed included *α*SMA, HSP27, ERK1/2, and GAPDH. While ideal densitometry data are directly proportional to protein abundance, our data confirm that densitometry data often deviate from this ideal, in which case they can fit nonproportional linear or hyperbolic mathematical models and can reach saturation. Nonlinear densitometry data were observed when Western blots were detected using infrared fluorescence or chemiluminescence, and under different SDS-PAGE conditions. We confirm that ghosting artefacts associated with overabundance of proteins of interest in Western blots can skew findings. We also confirm that when data to be normalised are not directly proportional to protein abundance, it is a mistake to use the normalisation technique of dividing densitometry data from the protein-of-interest with densitometry data from loading control protein(s), as this can cause the normalised data to be unusable for making comparisons. Using spiked proteins in a way that allowed us to control the total protein amount per lane, while only changing the amount of spiked proteins, we confirm that nonlinearity and saturation of densitometry data, and errors introduced from normalisation processes, can occur in routine assays that compare equal amounts of lysate. These findings apply to all Western blot studies, and we highlight quality control checks that should be performed to make Western blot data more quantitative.

## 1. Introduction

Western blotting is routinely used to detect proteins and their posttranslational modifications (PTM) in biological samples. The development and widespread uptake of modern imaging devices that capture digital images of Western blots, visualised through chemiluminescence or fluorescence detection, have resulted in the assumption of quantitative Western blotting in the literature. Each pixel in these digital images is assigned an intensity value that is related to the number of photons detected by the corresponding pixel in the sensor until it reaches saturation [1]. Non-detector saturated images are used for quantitation, and protein/PTM abundance is most often measured using an optical density (O.D.) algorithm, which calculates O.D. values from the background-corrected band intensity and band area of the protein/PTM of interest. All dedicated Western blot analysis software from major suppliers provides the ability to measure O.D. with minor vendor-vendor variations in implementation.

Ideal O.D. data are proportional to protein/PTM abundance and are therefore best modelled with linear regression through the origin (henceforth referred to as the directly proportional or proportional linear model, equation y = mx) [2–5]. It is commonly assumed that this model fits O.D. data [5], and only rarely are experimental data supporting this assumption included in published studies. Recent studies have, however, shown that O.D. data can violate this assumption and in these cases is better modelled with nonproportional linear functions (equation y = mx + b) or nonlinear hyperbolic functions [2, 4–6]. Identification of the appropriate model for O.D. data is critical because analysis of data using an inappropriate model results in incorrect estimation of the magnitude of any differences in protein/PTM abundance between samples [2, 4, 5, 7]. In extreme cases, Western blot data can become saturated (independent of saturation of the detection system), after which further increases in protein abundance cannot be detected and/or measured [2–21]. It is inappropriate to quantitatively analyse saturated Western blot data as differences in protein/PTM abundance between samples can be missed, resulting in false-negative findings [3, 4, 8, 12, 13].

Most quantitative Western blot studies normalise levels of target protein(s)/PTM(s) to levels of loading control protein(s) that do not change in abundance between comparisons [7, 9–12, 17, 18, 20, 22]. This normalisation method is used to correct for differences in protein abundance that are not relevant to the biological question being addressed [7, 9–12, 17, 18, 20, 22]. Normalisation is routinely performed by dividing the O.D. value of the target protein/PTM by the O.D. value of loading control protein(s) that were detected in the same sample [6, 10, 12, 13] and ideally were run in the same gel lane and detected in the same Western membrane [23]. This ratio (target/loading control(s)) is used for comparisons and any difference between samples is believed to correspond to the difference in target protein/PTM abundance. It is not widely recognised, however, that this normalisation strategy is only valid when O.D. data fit directly proportional models [4–6, 24]. Considering that in the best case scenario O.D. data only conform to directly proportional models over limited dilution ranges of lysate [2–21, 25], it is possible that many Western blot studies using this normalisation approach have been compromised by incorrect assumptions.

Western blots are among the most prevalent experimental tools used to study proteins in reproductive biology, which includes studies on myometrial smooth muscle function. In this field it is standard practice to normalise O.D. data of target proteins to loading control protein(s), such as alpha-smooth muscle actin (*α*SMA) [26–28], *β*-actin [29], glyceraldehyde 3-phosphate dehydrogenase (GAPDH) [30], and *α*-tubulin [31]. It is also common for PTM specific data to be normalised to total levels of the target protein [28, 32, 33]. We performed a random search of over 100 PubMed-indexed papers published between 2005 and 2017 that investigated myometrial function using quantitative Western blotting and found that only one [30] had presented Western blot validation data in either the main text or supplementary material.

Our study aimed to test whether quantitative Western blotting experiments, using untested assumptions of proportionality for normalisation, can be misleading and affect the study of human myometrial tissue proteins. Similar mistakes can befall all Western blot studies regardless of the sample type [2–21, 25]. While uncertain antibody specificity threatens the validity of any Western blot assay [2, 3, 20, 34–37], a Western blot performed using an antibody that does not recognise the correct target can only be fixed by replacing it with one that does. We therefore focus on possible errors that may occur during the quantification steps of Western blotting.

As part of these studies we comprehensively compared Western blot detection by chemiluminescence and infrared fluorescence and we provide an example of a quantitative Western blotting analysis using a proper workflow.

We hope that increased knowledge of the limitations of Western blot quantification will allow scientists to better use this technology to produce meaningful data and consequently lead to the routine inclusion of validation data in future publications.

## 2. Materials and Methods

### 2.1. Protein Extraction and Preparation

These studies were approved by the Hunter and New England Area Human Research Ethics Committee, adhering to guidelines of the University of Newcastle and John Hunter Hospital, Newcastle, Australia (02/06/12/3.13). Myometrial tissue biopsies were collected during caesarean section from term nonlabouring and term labouring women who had provided informed written consent. These samples were snap frozen in liquid nitrogen (LN_2_) and stored at -80°C. When processed, the samples were always stored on dry ice between steps until they were immersed in protein lysis buffer. Tissues were crushed in a metal mortar and pestle. Samples were placed into the mortar in a small pool of LN_2_ and crushed by hitting the pestle with a mallet immediately after the LN_2_ had evaporated. Following crushing, samples were immersed in LN_2_ then transferred to individual CK28-R 1.5 mL Precellys tubes (Cat. No. KT03961-1-007.2, Bertin Technologies). The mortar and pestle was cleaned with 70% ethanol and precooled with LN_2_ for each sample. When all samples were crushed, they were placed on ice and lysed in 1 mL of 2D extraction buffer (8 M Urea, 2 M Thiourea, 4% CHAPS) or 1 mL of SDS extraction buffer (2% SDS, 50 mM Tris pH 6.8, 5 mM EDTA). cOmplete mini-protease inhibitor cocktail tablets (Cat. No. 4693124001, Roche) and PhosSTOP phosphatase inhibitor tablets (Cat. No. 4906837001, Roche) were used in both lysis buffers. After addition of lysis buffer, the samples were immediately homogenised in a Precellys 24 homogeniser (Bertin Technologies) at 5000 rpm using 2 × 30 sec homogenisation intervals with a 20 sec break between each homogenisation. Samples were then stored on ice for 2 min to prevent sample warming and the Precellys homogenisation step was repeated twice more. Samples were then spun for 10 min at 16000*g* at 4°C to pellet insoluble cellular debris. Supernatant was extracted, transferred to fresh tubes, and stored at -80°C. The amount of protein in each sample was estimated using protein quantification assays run according to manufacturer's instructions. 2D extracts were quantified using the 2-D Quant Kit (Cat. No. 80-6483-56, GE Life Sciences). SDS samples were quantified using the BCA Protein Assay Kit (Cat. No. 23227, Thermo Fisher Scientific). Measurements were performed in a flat-bottomed 96-well plate at 480 nm wavelength for the 2-D Quant Kit and 562 nm wavelength for BCA Protein Assay Kit using a SPECTROstar Nano plate reader (BMG LABTECH). Pooled samples were created by mixing equal protein amounts (*μ*g of total protein) of individual samples that were extracted in the same lysis buffer. The protein concentration in pooled samples was reestimated using the appropriate quantification kit listed above for 2D or SDS buffer. Each individual sample as well as pooled samples was aliquoted and stored at -80°C.

### 2.2. Spiking of Myometrial Homogenates with Recombinant Proteins

Protein spiking experiments used pooled human myometrium homogenates made from 3 nonlabouring tissues that were extracted in 2D lysis buffer. Extraction of these samples, pooling, and protein quantification were performed identically to the 2D extractions described above. This pooled sample was diluted to 2 mg/mL in 1 mL of 2D lysis buffer. 900 *μ*L (1800 *μ*g) of this sample was used to reconstitute 100 *μ*g of purified recombinant ENPP1 (Cat. No. Ab167943, Abcam) and then 100 *μ*g of Fam3a (Cat. No. Ab167946, Abcam) and then made to a final volume of 1 mL. A subsequent total protein quantification assay (2-D Quant Kit run according to manufacturer's instructions) confirmed that the protein content was still approximately 2 mg/mL. This method of protein spiking created a sample in which each spiked protein constituted approximately 10% of the total amount of protein in the lysate. This sample was then serially diluted 1:1 with the 2 mg/mL unspiked pooled lysate to create eight 2-fold serial dilutions that contained decreasing amounts of the spiked proteins at similar concentrations of total protein. These samples were then aliquoted and stored at -80°C, and an individual aliquot was used for each membrane. Protein separation and Western blotting were performed using the same equipment as the SDS samples described below. Western blot signals for recombinant ENPP1 and Fam3a were simultaneously imaged in the same Western blot membranes using an anti-6x-His-tag antibody (see Supplementary Table S1 for details) that recognised the 6x-His-tag present on both proteins.

### 2.3. SDS-Polyacrylamide Gel Electrophoresis and Membrane Transfer

Stock aliquots of pooled myometrial tissue homogenates were defrosted on ice, warmed to room temperature, and then vortexed to ensure complete suspension of proteins. Samples were checked by eye to ensure that precipitates were not present. If precipitates were seen, the samples were left for 1 min at room temperature and then revortexed. This was repeated until samples became translucent. Samples were then stored on ice wherever possible, taking care to avoid the formation of precipitants. Each dilution of lysate was prepared as a stock sample with a volume of at least 10 *μ*L and an independently prepared stock sample was used for each membrane in which the same protein(s) were detected. Samples loaded into gels were made up to the same final volume using the necessary amounts of stock sample, lithium dodecyl sulphate (LDS) sample buffer, reducing agent, and lysis buffer. Each sample was heated at 70°C for 10 min, then centrifuged for 15 secs on a Heraeus Pico microfuge set to 16000*g*, and loaded into their respective gels. Whether each dilution series was serial or independent dilutions is listed in the figure legends. Novex Sharp Prestained Protein Standard (Cat. No. LC5800, Thermo Fisher Scientific), MagicMark XP (Cat. No. LC5602, Thermo Fisher Scientific), and SeeBlue Plus2 Prestained Protein Standard (Cat. No. LC5925, Thermo Fisher Scientific) were used for molecular weight sizing.

### 2.4. Separation and Protein Transfer to Membranes of 2D Samples That Did Not Contain Recombinant Proteins

All 2D samples that were not spiked with recombinant proteins were run on 12-well 1 mm thick 4-12% NuPAGE Bis-Tris gels (Cat. No. NP0322, Thermo Fisher Scientific). Each sample loaded consisted of lysate diluted in 2D lysis buffer and the appropriate amount of NuPAGE LDS Sample Buffer (Cat. No. NP0008, Thermo Fisher Scientific), and NuPAGE Sample Reducing Agent (Cat. No. NP0009, Thermo Fisher Scientific). Protein separation was performed immediately following gel loading at 200 V, 120 mA, and 25 W for 55 min in NuPAGE MOPS SDS Running Buffer (Cat. No. NP0001-02, Thermo Fisher Scientific). Proteins were then transferred onto Optitran BA-S 85 Reinforced Nitrocellulose (Cat. No. 10439196, GE Life Sciences) or Immobilon-PSQ Polyvinylidene Difluoride (PVDF) membrane (Cat. No. ISEQ00010, EMD Millipore) in 1× NuPAGE Transfer Buffer (Cat. No. NP0006-1, Thermo Fisher Scientific) containing 10% methanol. PVDF membranes were prewet in 100% methanol for 1 min before transfer. Proteins were transferred at 25 V, 120 mA, and 17 W for 1 h and 10 min. SDS-PAGE and transfer steps were performed in an XCell SureLock Mini Gel Tank (Thermo Fisher Scientific) on a Power Zoom-dual power pack or Power Ease 500 power pack (Thermo Fisher Scientific).

### 2.5. Separation and Protein Transfer to Membranes for SDS Samples, and 2D Samples Containing Recombinant Proteins

All SDS samples were run on 10-well 1 mm thick 4-12% Bolt Bis-Tris Plus gels (Cat. No. NW04120, Thermo Fisher Scientific). Each sample loaded into each gel consisted of lysate diluted in SDS lysis buffer and the appropriate amount of Bolt LDS Sample Buffer (Cat. No. B0007, Thermo Fisher Scientific) and Bolt Reducing Agent (Cat. No. B0009, Thermo Fisher Scientific). 2D samples containing recombinant proteins shown in Figure 4 were run on the same type of gels and transferred to the same membrane type using identical conditions. After loading the gels, proteins were separated at 165 V and 125 mA for 45 min in 1× Bolt MOPS SDS Running Buffer (Cat. No. B000102, Thermo Fisher Scientific), and then proteins were transferred to Odyssey nitrocellulose membranes (Cat. No. 926-31092, LI-COR Bioscience) at 10 V and 160 mA for 1 h using 1× Bolt Transfer Buffer (Cat. No.BT00061, Thermo Fisher Scientific) containing 10% methanol. All SDS-PAGE and Western blot transfer of these protein samples were performed in Bolt mini-gel tanks (Thermo Fisher Scientific) using PowerEase 90 W power packs (Thermo Fisher Scientific). To make the example Western blot shown in Figure 5, two samples containing recombinant ENPP1 and Fam3a proteins were diluted 1 in 10 with the labelling buffer supplied in an Amersham QuickStain kit (Cat. No. RPN4000, GE Life Sciences) to a protein concentration of 0.2 *µ*g/*µ*L. A standard curve was made by preparing a 2-fold serial dilution series of lysate from another sample containing these spiked-in recombinant proteins using the labelling buffer of the Amersham QuickStain kit as diluent (the protein concentrations of these standards ranged between 0.0125 and 0.4 *µ*g/*µ*L). 10 *µ*L of each sample (2 *µ*g of total protein for samples treated as unknowns, and between 0.125 and 4 *µ*g of total protein for standards) was combined with 6.25 *µ*L of Amersham QuickStain labelling buffer, 6.25 *µ*L of Bolt 4 × LDS Buffer, and 1 *µ*L of undiluted Cy5 stain from the Amersham QuickStain kit. The labelling reaction was performed for 30 minutes at room temperature in the dark before it was stopped by addition of 2.5 *µ*L of 10× Bolt Reducing Agent. All samples were then heated for 10 minutes at 70°C before they were pulse spun for 15 sec on a Heraeus Pico microfuge set to 10,000*g* and loaded onto a 12-well 1 mm thick 4-12% Bis-Tris SDS-PAGE Gel (Cat. No. NW04122, Thermo Fisher Scientific). After loading the gel, it was immediately run at 165 V and 125 mA for 50 min in 1 × Bolt MOPS SDS Running Buffer. Following SDS-PAGE, the gel was rinsed 3 × 30 sec in 1 × Bolt Transfer Buffer containing 10% methanol and then, after being wet with transfer buffer, it was fluorescently imaged using a GE Life Sciences AI600 set-up for Cy5 detection (approximately 10 minutes of image capture). The proteins were then transferred to Protran Reinforced nitrocellulose membrane (Cat. No. 10600016, GE Life Sciences) for 1 hour at 10 V, 165 mA, in 1 × Bolt Transfer Buffer containing 10% methanol. After the transfer step, the membrane was rinsed with MilliQ water, and Cy5-labelled proteins present on the membrane were fluorescently imaged using a GE AI600 (approximately 5 minutes of image capture). The membrane was rinsed in Tris-Buffered Saline with 0.1% Tween-20 (TBST) and blocked in 5% skim milk powder in TBST for 1 hour at room temperature. The membrane was then incubated with primary antibody (anti-6x-His-tag antibody described in Supplementary Table S1) diluted 1/1000 in 5% skim milk powder in TBST for approximately 18 hours at 4°C. The membrane was then washed 3 × 5 min in TBST and incubated with secondary anti-Mouse IgG, Horse Radish Peroxide (HRP)-conjugated antibody (described in Supplementary Table S1) diluted 1/3000 in 5% skim milk powder in TBST for 1 hour at room temperature. Before detection, the membrane was washed 3 × 5 min in TBST and cut horizontally in half, and the top half containing recombinant ENPP1 was developed by immersion in Luminata Classico Western HRP substrate (Cat. No. WBLUCO100, EMD Millipore) for 1 minute at room temperature. The bottom half containing Fam3a was developed by immersion in Luminata Forte Western HRP substrate (Cat. No. WBLUF0100, EMD Millipore) for 5 minutes at room temperature. After development, excess substrate was drained off and images were immediately captured on a AI600.

### 2.6. Total Protein Staining and Western Blotting

Except for the Western blot shown in Figure 5, for which the method used is described above, all other membranes were dried between filter paper overnight immediately after the transfer step. Before performing Ponceau S staining, PVDF membranes were immersed in 100% methanol for 1 min followed by MilliQ water for 1 min, while nitrocellulose membranes were immersed in MilliQ water for 1 min. Membranes were then stained for 5 min in 100 mL of Ponceau S solution (0.1% w/v Ponceau S (Cat. No. P3504-50G, Sigma-Aldrich) in 5% acetic acid). Blots were rinsed 3 × 1 min in 100 mL MilliQ water and imaged using GE Life Sciences LAS-3000 or AI600 Western imaging systems. Images captured on the LAS-3000 were taken using light illumination at 1/60 sec exposure. Images on the AI600 were captured using automatic exposure on colorimetric setting. Membranes were then destained with 6 × 5 min washes in TBST or phosphate buffered saline (PBS) for chemiluminescence and infrared detection, respectively. Any blots not stained with Ponceau S were rehydrated and put through the same destaining washes as blots that were stained. Blocking of the membranes for Western blotting was performed immediately after Ponceau S destaining. Western blotting experiments were conducted at room temperature using 1 h incubations for membrane blocking, 2 h incubations for primary antibodies, and 1 h incubations for secondary antibodies. Table S1 in supplementary materials contains the specific details on the antibodies used, conditions for blocking and primary antibody incubations for chemiluminescence detection, and antibody dilutions used. All membranes detected with infrared fluorescence were blocked in Odyssey blocking buffer (OBB) (Cat. No. 927-40000, LI-COR Bioscience), and all primary antibodies used on these membranes were diluted in OBB containing 0.1% Tween-20 to the same concentration as that used on membranes detected with chemiluminescence. In both methods, 3 × 5 min washes were conducted between primary and secondary antibody incubations as well as after secondary antibody incubation prior to imaging. Washes used TBST solution for chemiluminescence detection and PBS with 0.1% Tween-20 (PBST) for infrared detection. When using infrared detection, we performed all steps from the secondary antibody incubation in the dark. Membranes detected by chemiluminescence were developed by immersion in Luminata Classico Western HRP substrate for 1 min. Excess substrate was drained off and images were immediately captured on a LAS-3000 or AI600. Membranes for infrared detection were scanned on the LI-COR Bioscience Odyssey CLx imaging system. Both wet and dry membranes were scanned in the Odyssey CLx. Representative images of Western blot and Ponceau S staining data not shown in the main results figures are provided in supplementary materials (Supplementary Figures S2, S3, S4, S5, S6, and S8).

### 2.7. Image Capture Software and Image Formats

**Figure 1:**
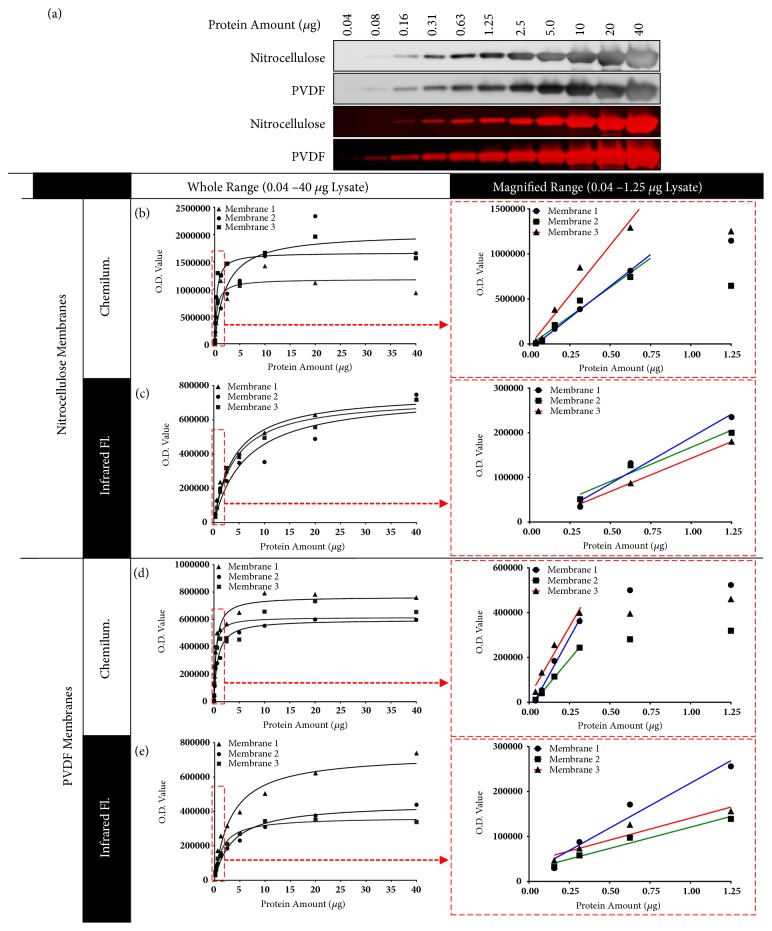
**Detection of commonly used loading control protein **α**SMA**. Pooled pregnant human myometrial tissue homogenates extracted in 2D lysis buffer were run in 2-fold serial dilutions from 40 *μ*g to 40 ng of total protein lysate per lane. (a) Representative Western blot images of membranes detected with chemiluminescence (black bands) or infrared fluorescence (red bands). O.D. data were calculated from band area and background-subtracted intensity for (b) nitrocellulose membranes detected by chemiluminescence, (c) nitrocellulose membranes detected by infrared fluorescence, (d) PVDF membranes detected by chemiluminescence, and (e) PVDF membranes detected by infrared fluorescence. The panels on the right side of subfigures (b-e) show the same O.D. data as each panel on the left but have been restricted to data obtained between 1.25 *μ*g and 40 ng of total protein lysate per lane to magnify the display of these data. The area that has been magnified in the left panels are shown by red rectangles. In these magnified regions, linear regression models are fitted to the data as shown by straight lines. 3 independently prepared membranes were analysed with each detection method.

**Figure 2:**
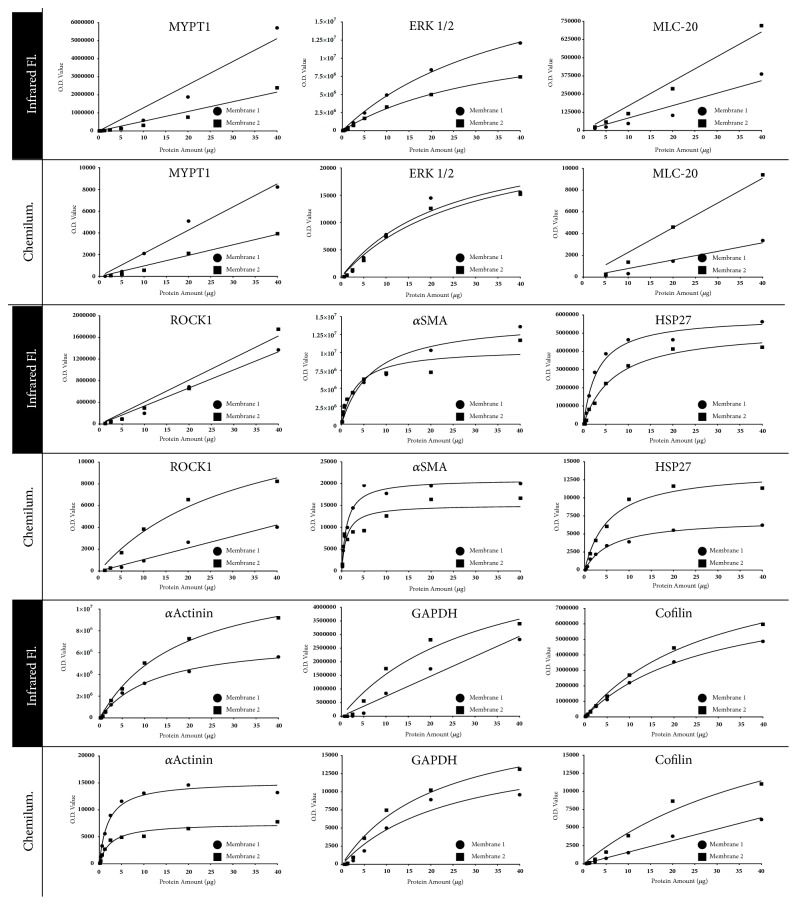
**O.D. data linearity is protein dependent**. Pooled pregnant human myometrial tissue homogenates extracted in an SDS-based lysis buffer were run in 2-fold serial dilutions from 40 *μ*g to 160 ng of lysate. The following proteins were detected (left to right from top to bottom): MYPT1, ERK1/2, MLC-20, ROCK1, *α*SMA, HSP27, *α*Actinin, GAPDH, and Cofilin. Each protein was detected with infrared fluorescence (top figure in each panel) and chemiluminescence (bottom figure in each panel). 2 independently prepared membranes were analysed with each detection method.

**Figure 3:**
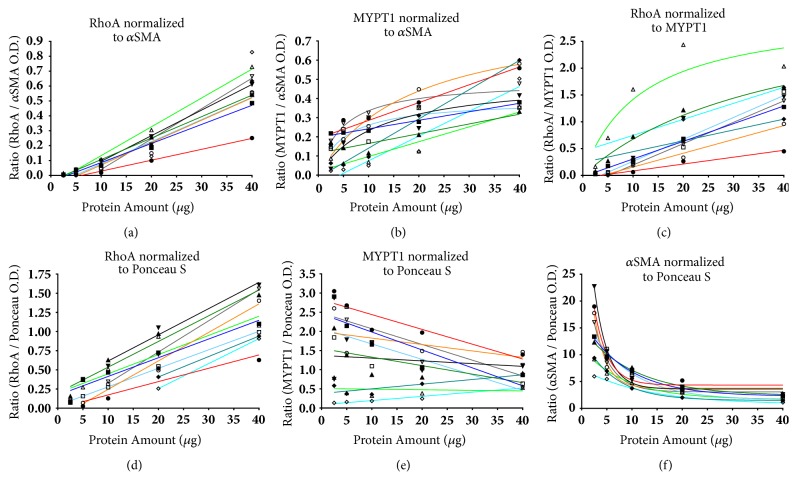
**Assessment of errors in normalisation of Western blot densitometry data**. Western blot O.D. data that were poor fits to proportional linear models were normalised to O.D. data from commonly used loading controls. Pregnant human myometrial tissue homogenates extracted in 2D lysis buffer were run in independent dilutions at 40, 20, 10, 5, and 2.5 *μ*g of total protein lysate. A myometrial sample from a woman not-in-labour and a sample from a woman in labour were run on each membrane and this was repeated five times with samples from different women used in each repeat. Densitometry was performed by selecting the highest non-detector saturated exposure that was available. These data were normalised to the indicated loading control from the same lane in each membrane. (a) RhoA normalised to *α*SMA, (b) MYPT1 normalised to *α*SMA, (c) RhoA normalised to MYPT1, (d) RhoA normalised to Ponceau S, (e) MYPT1 normalised to Ponceau S, and (f) *α*SMA normalised to Ponceau S.

**Figure 4:**
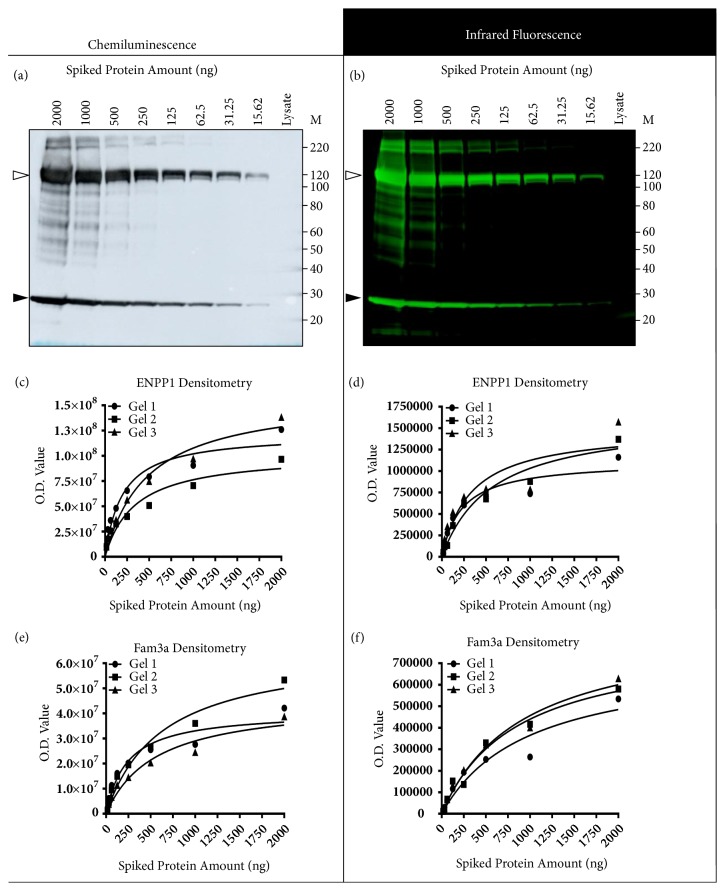
**Densitometry analyses and representative Western blots of lysates spiked with recombinant proteins**. (a) Representative blot detected using chemiluminescence. (b) Representative blot detected using infrared fluorescence. In both images the top band at approximately 120 kDa is recombinant ENPP1 (open arrow) and the bottom band at approximately 25 kDa is recombinant Fam3a (closed arrow). MagicMark XP was used for sizing (not shown). (c) ENPP1 O.D. values in membranes detected with chemiluminescence. (d) ENPP1 O.D. values in membranes detected with infrared fluorescence. (e) Fam3a O.D. values in membranes detected with chemiluminescence. (f). Fam3a O.D. values in membranes detected with infrared fluorescence. 3 independently prepared membranes were used for each detection method.

**Figure 5:**
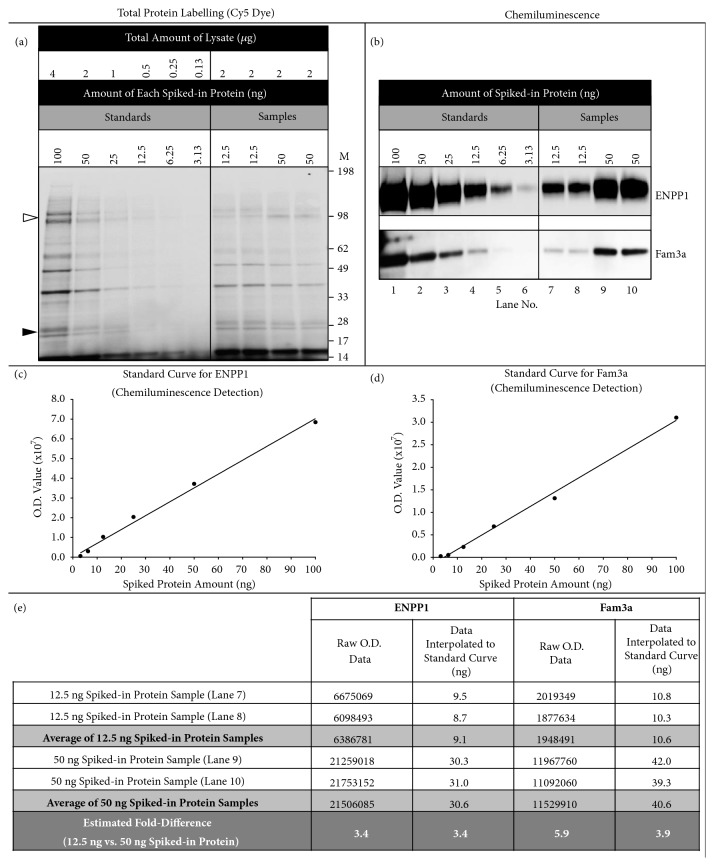
**Example of a Western blotting experiment that could be used to estimate differences in protein abundance**. (a) Fluorescent image of the membrane showing proteins in each lysate that were labelled with Cy5. SeeBlue Plus2 Marker was used for sizing (not shown). This image was used to estimate the total protein abundance for each sample. Arrows indicate the approximate location of ENPP1 (open arrow) and Fam3a (closed arrow). (b) Western blot images of recombinant ENPP1, and Fam3a that were used to compare their abundance. These proteins were detected by chemiluminescence. (c) The standard curve of recombinant ENPP1 O.D. values that was used to interpolate levels of recombinant ENPP1 in each sample. (d) The standard curve of recombinant Fam3a O.D. values that was used to interpolate levels of recombinant Fam3a in each sample. (e) This table shows the results of interpolating the O.D. data against the standard curve for that protein as well as the comparisons made using the raw O.D. data.

Images captured using the LAS-3000 system were taken in Fujifilm Image Read LAS-3000 software version 2.0 and saved as raw.inf/.img files. AI600 images were taken in GE Imager Version 1.2.0 and saved as TIFF files. Westerns scanned using the Odyssey CLx were imaged using LI-COR Image Studio software version 2.1.10 and images were saved as a work area. All densitometry analyses were performed on images saved in the above formats. Images of colorimetric Ponceau S stains as well as Western blots detected with chemiluminescence, which were analysed as part of the datasets presented in Figures 1–4, were analysed using MultiGauge image analysis software version 3.0. The example Western blot data and Cy5-total protein labelling data presented in Figure 5 were analysed using Image Quant TL software version 8.1. All densitometry analyses performed on membranes scanned on the Odyssey CLx was done using Image Studio Light version 5.

### 2.8. Densitometry Method

In all software packages the region of interest (ROI) encircling each band was defined manually. To ensure that the entire band was captured, the values in the lookup table were adjusted to increase contrast. This does not alter the underlying values for quantification. All bands at the correct molecular weight ± approximately 5 kDa were analysed as the signal for that target protein. In this region any overlapping visible bands in both the Western blot image and/or electrophoretogram were included to ensure that the level of background signal subtraction was appropriate to the level of background noise in the membrane and was not artificially affected by detected signal. As comparisons are made against the same sample lysate, any nonspecific overlapping bands that may be present in this region are considered to be of constant relative abundance and are unlikely to interfere with data interpretation. Any other bands visible at different molecular weights were excluded from analyses. When bands in adjacent lanes were touching due to high protein load, the boundaries of the of ROI were placed at the point of minimum thickness between the bands. The densitometry data for Ponceau S total protein stain images were obtained from all proteins visible in each entire lane. All data normalisation processes were performed by dividing the O.D. value of the target protein by the O.D. value of the chosen loading control.

### 2.9. Analyses Using MultiGauge Software

Images were analysed with MultiGauge software using automatic horizontal or polygonal baseline detection with settings H ratio 10% and V ratio 70%. The width of a band was defined manually. The limits of the band were chosen to be the region on each side of the curve (top/bottom of band) in which the line denoting intensity either touched or ran parallel with the line denoting baseline.

### 2.10. Analyses Using Image Quant TL Software

The rolling ball algorithm set to a radius of 50 pixels was used for background subtraction. The limits of each protein band were chosen to be the region on each side of the curve (top/bottom of band) in which the line denoting band intensity touched the line denoting baseline.

### 2.11. Analyses Using Image Studio Software

Densitometry performed in Image Studio used median local background correction with a border width of 1 unit. The location of the region for background correction was defined around the adjacent pixels of the ROI using the top/bottom only, right/left only, or the entire ROI setting. This selection was changed to prevent counting (as background) the signal from overlapping between neighbouring lanes as well as any nontarget bands 5-10 kDa away from the target protein molecular weight as these were likely to interfere with proper background subtraction if unaccounted for. Once the border region was chosen for background subtraction, this selection was kept consistent across all repeats analysing the same protein.

### 2.12. Statistical Analysis of Regression Fits

Regression modelling was performed using GraphPad Prism version 6. All regression models were fit using the least squares method. Comparisons between the fit of each dataset by hyperbolic and linear regression models were made using the extra-sum-of-squares F-test with an* F*-statistic threshold of *p* = 0.05 for model selection [38].

## 3. Results

### 3.1. O.D. Data May Be Saturated When Western Blots Are Analysed at Typically Used Amounts of Protein Lysate

2-fold serial dilutions of pooled lysates of term pregnant human myometrial tissue were separated by SDS-PAGE and transferred to nitrocellulose or PVDF membranes. These were probed with an anti-*α*SMA antibody and detected using chemiluminescence or infrared fluorescence (Figure 1). O.D. data were analysed for saturation by comparing a hyperbolic model against a linear model [4].

O.D. data from all membranes were better fit with hyperbolic rather than linear models, indicating that data were becoming saturated as the amount of lysate loaded into the gels increased (Figures 1(a)–1(e)). The linear region of detection occurred below approximately 0.3-1.25 *μ*g of lysate. In the linear region of detection, O.D. data directly correspond to protein abundance only if the data fit directly proportional linear models [2, 5]. If linear O.D. data instead fit nonproportional linear models, the O.D. data do not directly correspond to protein abundance, and if this is not accounted for, any estimates of protein abundance differences between samples will be incorrect [2, 5]. In these Western blots, O.D. data above approximately 0.3-1.25 *μ*g of lysate were nonlinear and plateaued when more than 5-20 *μ*g of lysate was analysed. This differed depending on the type of membrane that was used. High dose hook-like effects were present in the plateaued region in all membranes detected by chemiluminescence. The nonlinear but not plateaued region of detection should only be used for quantification if O.D. data from samples are interpolated against O.D. data of a standard curve that has been produced from a calibrator sample (lysate or purified protein) run over enough dilutions to define the appropriate model [2, 4, 5, 21, 25]. This method can also be used to estimate protein abundance differences when O.D. data are linear but nonproportional [2, 5]. To control for membrane-specific effects, it is important that the dilution series of the calibrator is included on each membrane that contains samples being compared [2, 4, 5, 21, 25], and O.D. data that fall outside the range of the standard curve should not be analysed [4, 20]. Only qualitative comparisons showing the presence or absence of a protein/PTM should be made in the region of detection where O.D. data have plateaued because protein/PTM abundance differences may not be detected [3, 4, 8, 12, 13].

Visual inspection of the Western blots (Figure 1(a)) shows that band area increases with protein load. This suggested that saturation of O.D. data might be attributable to the measurement of band intensity and, therefore, that measuring band area alone may provide a solution to the saturation problem. For blots detected with chemiluminescence, we separately examined band intensity (Supplementary Figure S1) and band area (Supplementary Figure S1). These data were both best fit with hyperbolic curves, indicating that neither measure alone surmounts the problem of O.D. data saturation.

### 3.2. Band Ghosting Artefacts Prevent Accurate Quantitative Comparisons

In Figure 1(a), band fading, known as “ghosting” [12, 21], can be seen above approximately 2.5 *μ*g of lysate in nitrocellulose membranes and 10 *μ*g of lysate in PVDF membranes detected with chemiluminescence. Ghosting was not observed in membranes detected with infrared fluorescence. This artefact results in bands appearing washed out [4, 21] and caused the aforementioned high dose hook-like effects in *α*SMA O.D. values that were present in Figures 1(b) and 1(d). Western blots that show ghosted bands are therefore unsuitable for quantitative comparisons because densitometry will not provide reliable measures of protein abundance [4, 21]. Since ghosting is associated with overabundance of the target protein, it should be addressed by reducing the amount of lysate that is loaded into SDS-PAGE gels [4].

### 3.3. Each Protein in a Sample Can Have Different Linearity

We investigated whether saturation affected the linearity of O.D. data for other myometrial proteins (Figure 2). As part of this experiment we also tested whether saturation occurred under different experimental conditions to those used for the samples shown in Figure 1. This was achieved in the analyses of the samples shown in Figure 2 by using a different human myometrial tissue lysate, a different SDS-PAGE system, supplier of nitrocellulose membrane, and chemiluminescence detection system.

Each protein analysed had different linearity and/or detectable range across the dilution series (40–0.16 *μ*g of lysate) (Figure 2). O.D. data for *α*ACTININ, *α*SMA, HSP27, and ERK1/2 were affected by saturation as they were fit with hyperbolic models. MYPT1 and MLC-20 O.D. data were unaffected by saturation since they fit linear models. ROCK1, Cofilin, and GAPDH O.D. data from at least one membrane per protein were fit by hyperbolic models, and at least one membrane for each protein was fit with linear models, which indicated that the fitting regression model can be different between membranes that contain repeats of an experiment. These differences between experimental repeats may not be under control of the researcher and could be due to differences in transfer efficiency that affected the amount of proteins present on these membranes. Detection by chemiluminescence or infrared fluorescence imparted only minimal differences in the models that fit O.D. data obtained for the same protein.

### 3.4. Your Normalised Western Data May Be Misleading

In a different set of experiments that used dilution series of different myometrial lysates, each membrane was stained with Ponceau S to detect total protein and then probed for MYPT1, *α*SMA, and RhoA. Proportional linear models were not appropriate fits for most MYPT1, *α*SMA, and RhoA O.D. data (Supplementary Figure S7). We were therefore able to explore under real experimental conditions whether data normalisation is compromised when O.D. data deviate from proportional linear models (Figure 3).

When we normalised one of these proteins against another protein (Figures 3(a), 3(b), and 3(c)) or a protein against the Ponceau S stain (Figures 3(d), 3(e), and 3(f)), the normalised ratio was not constant, and it systematically differed across each dilution series in a manner that was specific to the proteins under investigation. These findings confirm that it is inappropriate to normalise target protein O.D. data by dividing it by the O.D. data of loading control protein(s) if the O.D. data do not fit proportional linear models [2, 7, 20, 24].

### 3.5. O.D. Data Nonlinearity and Normalisation Error Can Occur under Typical Western Blot Conditions

We spiked myometrial homogenates with two purified recombinant proteins, ENPP1 (~97 kDa) and Fam3a (~23 kDa). By accounting for the amount of each recombinant protein and using the unspiked lysate as a diluent, we were able to vary the concentrations of spiked-in proteins in a 2-fold serial dilution series while maintaining similar total amounts (w/v) of lysate. These samples allowed us to assess O.D. data linearity and normalisation error under conditions like how typical Western blot comparisons are performed, in which samples are compared at a single amount of lysate that is held constant between comparisons.

O.D. data from ENPP1 and Fam3a were both fit with hyperbolic models (Figure 4). There was only a small range of ENPP1 and Fam3a O.D. data that were approximately linear, which occurred below approximately 250 ng of spiked-in protein (<0.63% of spiked-in proteins in the total lysate). In each sample lane, we normalised ENPP1 to Fam3a and separately normalised ENPP1 and Fam3a to Ponceau S O.D. data (Supplementary Figure S9). When ENPP1 was normalised to Fam3a (this ratio should be constant between samples), a small normalisation error of approximately 2-fold was found across the dilution series. The Ponceau S O.D. data were similar between lanes (Supplementary Figure S8), which was expected because each sample had a similar amount of lysate loaded into the gel; hence normalisation of ENPP1 and Fam3a to Ponceau S was like dividing the raw ENPP1 and Fam3a O.D. data by a constant, and the normalised data preserved the hyperbolic pattern of the raw data. If these normalised data (ENPP1:Ponceau S or Fam3a:Ponceau S) were used to compare ENPP1 or Fam3a protein levels between these samples, the estimated differences in protein abundance would be incorrect. All normalisation errors observed in this dataset were similar in pattern and magnitude between membranes detected with chemiluminescence or infrared fluorescence.

### 3.6. More Accurate Estimation of Differences in Protein Abundance May Be Made after Calibration of Western Blotting Experiments

Figure 5 shows an example of how quantitation of protein abundance using Western blot O.D. data can be successfully performed. Using the data from the experiments in Figure 4 as the guide, we performed an experiment in which we compared, in duplicate, two samples that had a 4-fold difference in the abundance of spiked-in recombinant ENPP1 and Fam3a proteins. The absolute amounts of recombinant ENPP1 and Fam3a protein analysed (12.5 ng in one sample vs. 50 ng in the other) were calculated to be within the linear range of detection, and we included a standard curve covering the range of 3.25–100 ng of recombinant ENPP1 and Fam3a spiked into myometrial lysate. Comparison of the total protein abundance on the membrane (used as a loading control) suggested that it did not differ by more than approximately 0.1-fold between the samples being compared (Figure 5 and Supplementary Table S2). This meant that it was unlikely that confounding factors affected comparisons. Thus, it would have been superfluous to put the Western blot O.D. data through a normalisation process. O.D. data for recombinant ENPP1 measured in the standards fitted a proportional linear equation (Figure 5) (equation y = 702615x, relative R^2^ = 0.993). The standard curve is useful here as a quality control check to demonstrate that the O.D. data fit this equation [2, 3, 7]. However, in this ideal situation [2–5], the standard curve does not improve estimations of differences in protein abundance. Thus, a 3.4-fold difference in recombinant ENPP1 protein abundance was found between the two samples being compared when comparisons were made using the raw O.D. data as well as when comparisons were made after O.D. data were first-interpolated against the standard curve. In contrast, O.D. data for Fam3a measured in the standards were better fit with a nonproportional linear equation (Figure 5) (equation y = 318398x – 1411334, R^2^ = 0.996) than a proportional linear equation (*P *= 0.0433, compared using extra-sum-of-squares F-test). A 5.9-fold estimated difference in recombinant Fam3a abundance was found between the two samples when the raw O.D. data were compared. As described above and highlighting the importance of the standard curve in these cases [2, 5], interpolation of the Fam3a O.D. data against the standard curve before comparisons were made resulted in a more accurate 3.9-fold difference in recombinant Fam3a abundance.

## 4. Discussion

Our study confirms that quantification of Western blot data is not straightforward [2–4, 20]; users cannot arbitrarily select the amount of lysate to load into a gel and expect meaningful results, even when protein loading is held constant across gel lanes.

Reproducibility, detection sensitivity, data linearity, and proportionality as well as the dynamic range of detection are important parameters to assess for any Western blot method [2, 3, 20]. When we compared these parameters between detection by chemiluminescence or infrared fluorescence, we did not find differences that were large enough to recommend that one method should be used over the other. While this is in conflict with some recent studies that suggested that detection using infrared fluorescence produced superior quantitative Western blot data [4, 18], our findings confirm that it is more useful to recognise that limitations exist in the collection and analysis of all Western blot data, regardless of the detection method [3]. Thus, only with proper quality control strategies can the interpretation of quantitative Western blot data be relied upon to have biological relevance [2–5, 7, 11, 20, 21, 25].

It is critical to address detector-independent saturation that affects the linearity of Western blot O.D. data in order to ensure that meaningful results are obtained [2–21]. Multiple explanations for this type of saturation have been suggested, including that it may be due to:

1. saturation of membrane binding sites [5, 11, 12], after which proteins will bind in layers that hide the detection of proteins layered underneath [11];
2. limited accessibility of dyes, antibodies, and/or antigens [4, 12];
3. the concentration of primary or secondary antibodies used [6, 25];
4. limitations in the local concentration of HRP substrate [25];
5. oxidation of HRP attached to the secondary antibodies [4, 20];
6. proximity quenching of infrared fluorescence reactions [4].

Regardless of the cause of saturation, our work and the work of others [2–21, 25] indicate that it should not be assumed that O.D. data provides a direct measure of protein abundance [2, 4, 5, 7]. Linear proportionality should not be assumed even when based on similar studies, as differences in methodology that appear trivial affect the linearity of O.D. data [4, 25]. Therefore, to allow proper data interpretation and increase the confidence that findings are accurate and reproducible, the appropriate model for O.D. data should be established and presented for every protein analysed in every study [2, 3, 7, 11, 12, 20, 39]. This assessment must be performed under identical conditions to those used to analyse experimental samples, and it is also essential that the levels of all proteins being studied are similar between the lysate used for this assessment and the experimental samples [3, 7, 11, 12, 20]. This may be accomplished using dilution series of pooled lysate from all of the experimental samples [3, 7, 11, 12, 20, 39]; however, because this method averages protein levels between samples, it should be ensured that the analysis is conducted across a dilution span that is sufficient to encompass all protein amounts that may be encountered in the study [11, 39].

O.D. data are commonly nonlinear and often plateaued when high abundance proteins (often housekeeping proteins such as GAPDH, *α*-Tubulin, and *β*-Actin) were detected above 1-10 *μ*g of lysate [2–21]. Our results, showing that *α*SMA and HSP27 O.D. data were nonlinear when more than approximately 1.25 *μ*g of human myometrial tissue lysate was analysed, further question the reliability of Western blot studies that have used other high abundance proteins as loading controls. It is worth restating that it is known that GAPDH, *α*-Tubulin, and *β*-Actin are often not appropriate loading controls, as their levels change under different biological and experimental conditions at the mRNA and/or protein level [7, 18, 40–45]. These changes in abundance may be due to* in vivo* biological differences [18], may be induced by the experimental treatments under investigation [7, 41], or may be related to the* in vitro* biology of experimental models [43]. Levels of cell-type specific proteins that are used as loading controls may also change under different experimental conditions. For example, Campbell et al. [45] found that *α*SMA mRNA levels changed according to cell density in primary cultured rabbit aortic smooth muscle cells. Therefore, it needs to be confirmed that levels of loading control protein(s) do not change between the experimental populations being compared or, if this evidence has already been published, it should be referred to [2].

We confirmed that when O.D. data deviate from proportional linear models, the reliability of normalisation performed by dividing target protein O.D. data with loading control protein O.D. data is compromised [2, 7, 20, 24]. Our data and that of others [7, 24] show that normalisation performed under these circumstances does not account for irrelevant differences in protein/PTM abundance regardless of whether these are biologically driven or due to technical artefacts. At best it will add variance to protein/PTM abundance estimates [12] and will lead to over-or-under estimation of the magnitude of protein/PTM abundance changes [2, 4, 5, 7, 24], and at worst it can produce normalised ratios that suggest that protein/PTM abundance differs in the opposite direction to what actually occurs in the samples being analysed. Therefore, normalised data obtained under these circumstances cannot be relied upon and must be interpreted with caution as it can lead to incorrect conclusions being made [2, 20]. It is difficult to quantitatively establish when O.D. data deviate enough from proportional linear models to make this normalisation strategy unusable. Hence, we suggest that whenever this normalisation method is used (when O.D. data are approximated by proportional linear models), the investigator must demonstrate that normalisation error cannot explain their findings and the data used to reach this conclusion should be shown. In situations where nonproportional O.D. signals are detected and the data from the protein-of-interest is to be normalised to one-or-more loading control(s), the nonproportionality should be accounted for, which may be achieved using alternative normalisation strategies [24] or by interpolating data against standard curves [2, 4, 5, 21, 25].

Total protein labelling methods are commonly recommended in the literature [2, 3, 7–10, 12, 13, 15, 17, 18, 20, 22, 25, 39]. These methods include stain-free technology that labels proteins within the gel that is subsequently used for Western transfer [2, 8, 9, 39], and stains such as Ponceau S that labels proteins bound to Western membranes [8, 15, 25]. Total protein labelling can be an appropriate loading control and O.D. data obtained from total protein labelling often remain linear at lysate loads greater than 5-10 *μ*g of protein [2, 7–9, 12, 15, 17, 18, 20, 25, 39]. It is important to consider whether total protein labelling interferes with subsequent immunodetection [9, 17]; however, this has only rarely been found [10] and is easily tested for by comparing simultaneously imaged Western blots containing identical samples, in which total protein labelling was performed for one membrane but not the other [7, 10, 12]. Unless total protein labelling has been shown to interfere with immunodetection, there are two important reasons why every Western blot membrane should be labelled for total protein and these data presented even when loading control protein(s) are also detected using antibodies:

1. Most types of proteins found in cell and tissue lysates are visualised by total protein labelling methods and, therefore, these techniques allow the levels of expression of many different proteins to be estimated [9, 10, 15, 20]. In contrast, immunodetection of specific loading control proteins is currently too resource intensive to allow routine detection of more than a few proteins on each membrane; thus, immunodetection has more chance of sampling error [9, 10, 15, 20]. Reporting of total protein labelling data can often allow other researchers to best answer questions on whether different samples have similar SDS-PAGE protein separation profiles and, when appropriate, whether the amount of total protein analysed for each sample is similar [9, 10, 15, 20].
2. Total protein labelling of Western membranes best shows artefacts such as degraded samples, smeared bands due to protein precipitation in the gel, and transfer errors such as air bubbles [7, 25].

It is also important to recognise that labelling all proteins in a sample does not discriminate between the sources of protein when more than one biological source is present [3]. Therefore, even if the same amount of total protein is detected between samples, it may originate from differences in cell types and/or extracellular matrix, and if these differences are not relevant to the experimental question being addressed, it can cause incorrect results [3]. While the same limitation applies for ubiquitously expressed loading control proteins detected using antibodies, this can occasionally be circumvented when examining a tissue-specific protein by using a loading control protein specifically expressed in the same tissue as the target protein [3]. To show that differences in tissue composition do not affect findings made by Western blotting when total protein labelling or ubiquitously expressed loading control proteins are used, a histological analysis should also be performed or the relevant literature should be cited [3].

## 5. Conclusions

We showed that there are many technical challenges to overcome when using Western blotting to compare the abundance of proteins found in myometrial tissue lysates. Our data support the literature and confirm that it is difficult to convert Western blotting from a qualitative technique to a quantitative technique [2–4, 7, 11, 20]. By analysing myometrial proteins including *α*SMA, HSP27, *α*Actinin, and GAPDH, we confirmed that loading too much lysate into an SDS-PAGE gel can cause problems such as detector-independent O.D. data saturation, nonlinearity of O.D. data, and ghosting artefacts in subsequent Western blotting experiments [2–4, 7, 11, 20]. Compounding these problems is the limitation that the standard method of data normalisation that is used in the field only works when the O.D. data used in the normalisation calculations are directly proportional to the abundance of the corresponding proteins in the samples under study [4–6, 24]. Our data confirmed that when this is not the case, confounding differences in protein abundance arising from technical or biological origin may not be accounted for in the normalised data. In conclusion, our findings, taken together with those in the literature [2–4, 7, 11, 20], suggest that much of the Western blot data being routinely reported in the scientific community are likely to be severely compromised and are unjustly being used to support claims of biological significance. Below we have made general recommendations based on those in the literature [2–4, 7, 11, 12, 20, 25, 39]. The use of these or similar recommendations by the scientific community will lead to a much higher quality standard of Western blot data that are reported.

*Recommendations*

1. Perform total protein labelling and image the labelled membranes. Present these images alongside all Western blot data or in supplementary material.
2. Densitometry analysis should not be performed on bands showing ghosting as it is unreliable and can lead to incorrect data interpretations.
3. Degraded samples showing band smearing, overloaded lanes with streaked bands due to protein precipitation in the gel, or bands with a wavy pattern indicative of overheated gels should not be used for quantitative comparisons.
4. Perform Western blot assays specifically designed to establish the dynamic range of detection and determine whether O.D data fit proportionally linear, linear but nonproportional, or nonlinear models for all target protein(s)/PTM(s) being assessed, as well as all loading control(s). This critical assessment should be performed under identical technical and sample conditions to those that will ultimately be used in the study. This is ideally repeated over at least two membranes to minimise the chance that membrane-specific effects interfered with this important validation step.
5. Load an experimentally justified amount of lysate. This amount should be based on calibrations performed in step (4). Quantitative comparisons should not be made in the saturated range of protein loading. If O.D. data fit linear but nonproportional or nonlinear models, this should be accounted for in all quantitative comparisons.
6. Western blots on validated unchanging loading controls should be run at optimised nonsaturated conditions. This may require samples investigating loading control(s) to be run at different dilutions in different lanes and/or membranes to target proteins.
7. Provide evidence that data normalisation steps are functioning for their intended purpose and are not introducing errors into the analysis.
8. Use appropriate statistical analyses and statistical blocking strategies that account for inter- and intramembrane differences.

## Data Availability

All densitometry data and representative images of Western blots and membranes labelled with total protein stains that were used to support the findings of this study are included within the supplementary materials.

## Supplementary Materials

Supplementary 1 Supplementary Table S1: This file contains details on the antibodies used in this study including supplier details, catalogue and lot numbers, the dilution of each antibody that was used, membrane blocking conditions, and antibody incubation conditions.

Supplementary 2 Supplementary Figures S1-S9: This file contains representative images of Western blot and Ponceau S staining data from membranes that had densitometry performed. It also contains supporting densitometry data and/or normalised data from Western blots that were used in the main figures.

Supplementary 3 Supplementary Table S2: This file contains the raw densitometry data and normalised data that was used in this study.
