# Supplementary material for: Misleading Westerns: Common Quantification Mistakes in Western Blot Densitometry and Proposed Corrective Measures
Source: Biomed Res Int. 2019 Jan 21;2019:5214821. doi: 10.1155/2019/5214821 (PMC6360618; doi:10.1155/2019/5214821)
Supplement: Supplementary 2 — Supplementary Figures S1-S9: This file contains representative images of Western blot and Ponceau S staining data from membranes that had densitometry performed. It also contains supporting densitometry data and/or normalised data from Western blots that were used in the main figures. [file 5214821.f2.pdf]

Supplementary Figures

Title: Misleading Westerns: Common Quantification Mistakes in Western Blot Densitometry and Proposed Corrective Measures.

Authors: Trent A.J. Butler[1,2*], Jonathan W. Paul[1,2], Eng-Cheng Chan[1,2], Roger Smith[1,2,3] and Jorge M. Tolosa[1,2]

[1] Mothers and Babies Research Centre, School of Medicine and Public Health, Faculty of Health and Medicine, University of Newcastle, Callaghan, NSW, Australia, 2308
[2] Hunter Medical Research Institute, 1 Kookaburra Circuit, New Lambton Heights, NSW, Australia, 2305
[3] John Hunter Hospital, New Lambton Heights, NSW, Australia, 2305

***Corresponding Author:** Trent A.J. Butler, Hunter Medical Research Institute, New Lambton Heights 2305, NSW, Australia.
Email: Trent.Butler@newcastle.edu.au

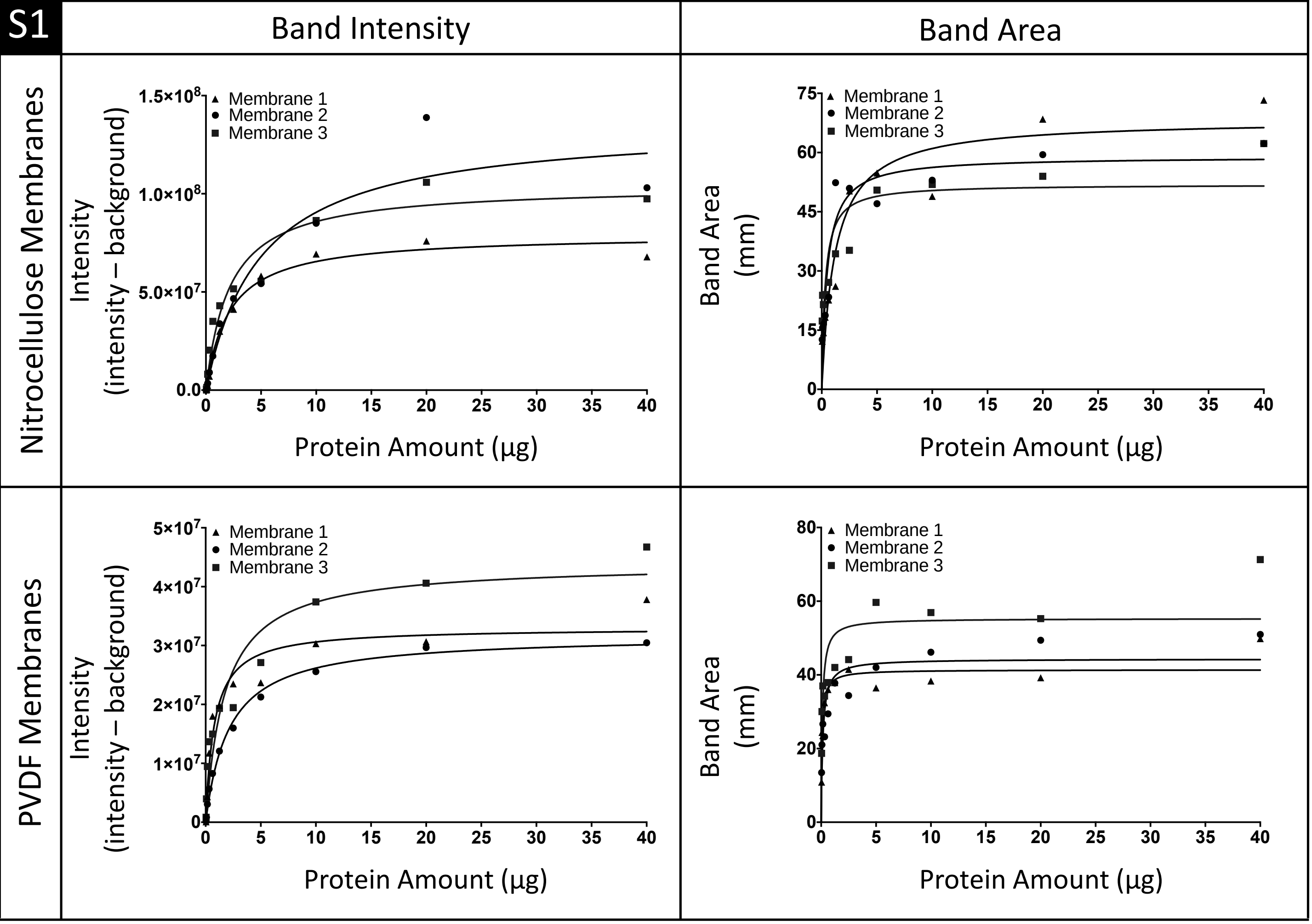

Supplementary Figure S1: Band Intensity and Band Area Measurements of the Membranes Used in Results Figure 1 That Were Detected by Chemiluminescence.

S2

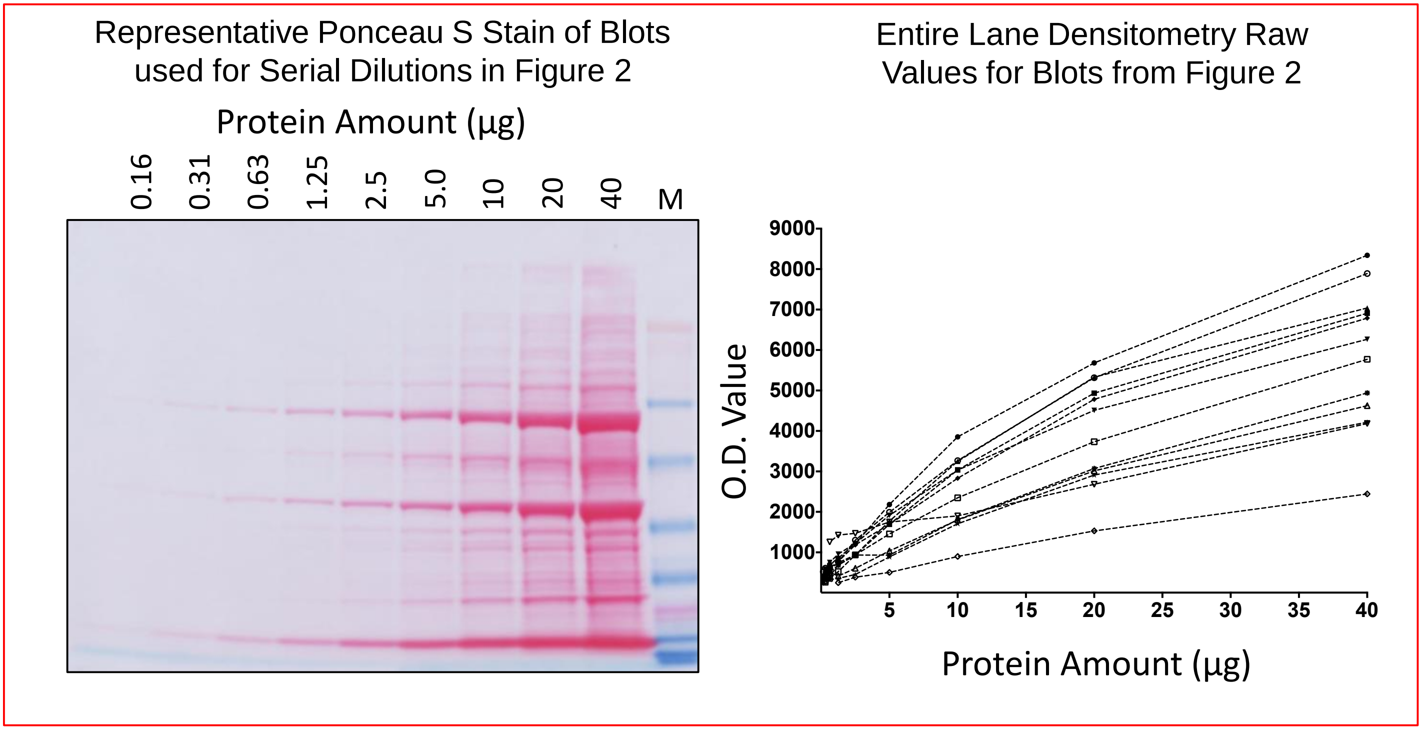

Supplementary Figure S2: Ponceau S Staining of the Membranes from Results Figure 2. The membranes were stained with Ponceau S and imaged on a GE AI600. Membranes detected with chemiluminescence and infrared fluorescence were stained. These data show a representative image of the Ponceau S stained membranes (left) and the quantified densitometry measurements made over the entire lane (right). In the right panel each connected line is the data from a membrane. The molecular weight marker was SeeBlue Plus 2 mixed 2:1 with MagicMark XP.

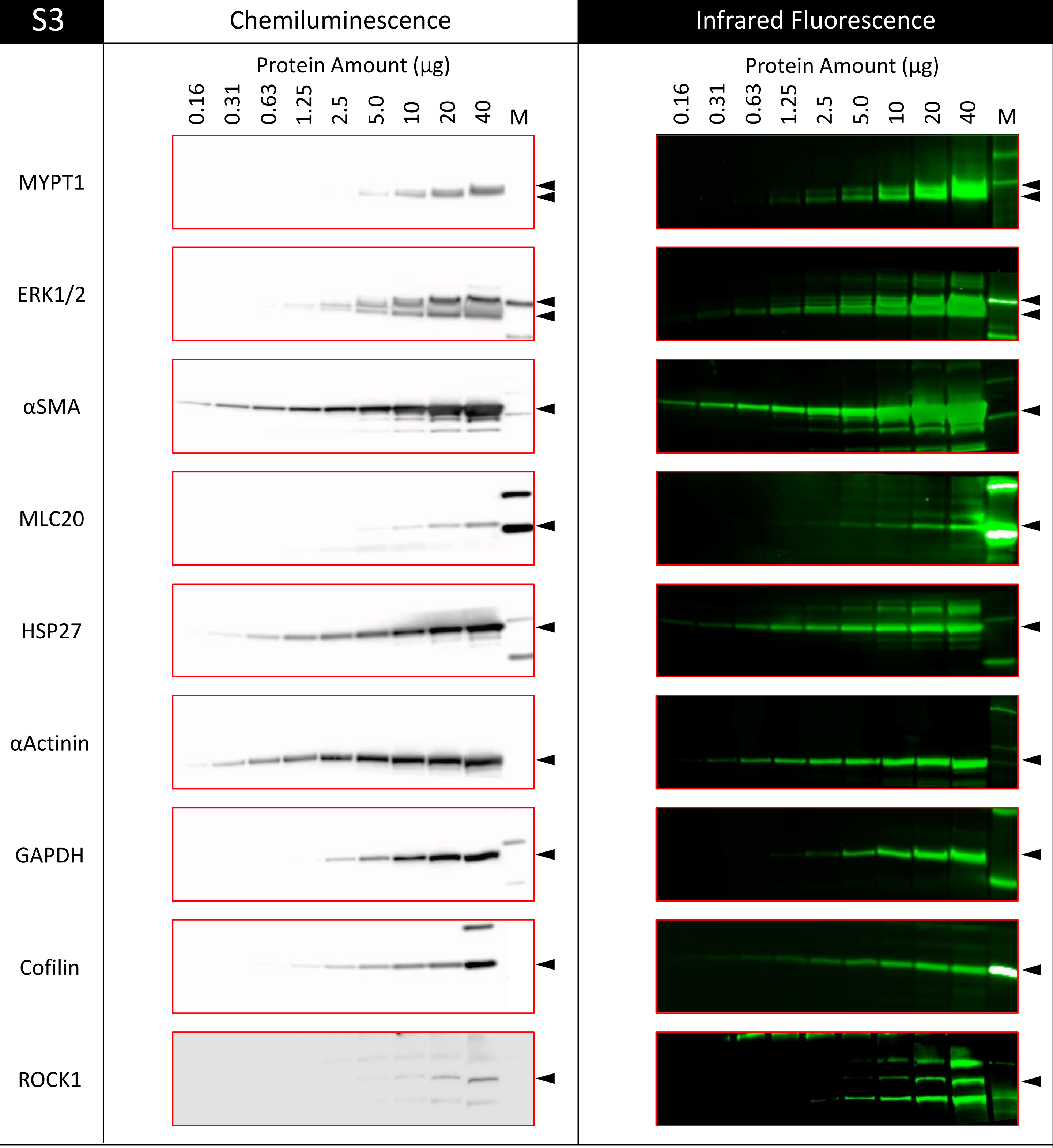
S3
Chemiluminescence
Infrared Fluorescence
Protein Amount (µg)
0.16
0.31
0.63
1.25
2.5
5.0
10
20
40
M
Protein Amount (µg)
0.16
0.31
0.63
1.25
2.5
5.0
10
20
40
M
MYPT1
ERK1/2
αSMA
MLC20
HSP27
αActinin
GAPDH
Cofilin
ROCK1

Supplementary Figure S3: Representative Western Blot Images of the Membranes from Results Figure 2. These data show the bands (arrows) that were analysed by densitometry. The following proteins were analysed. MYPT1, ERK1/2, αSMA, MLC20, HSP27, αActinin, GAPDH, Cofilin and ROCK1. For MYPT1 and ERK1/2, which showed the presence of two major bands consistent with their known isoforms, both bands were analysed as a single unit as the SDS-PAGE resolution was insufficient to properly separate them. Additional bands falling outside the expected molecular weight were excluded from the analysis. Left panel- Membranes detected with chemiluminescence. Right panel – Membranes detected with infrared fluorescence. The odyssey membranes were dried before scanning.

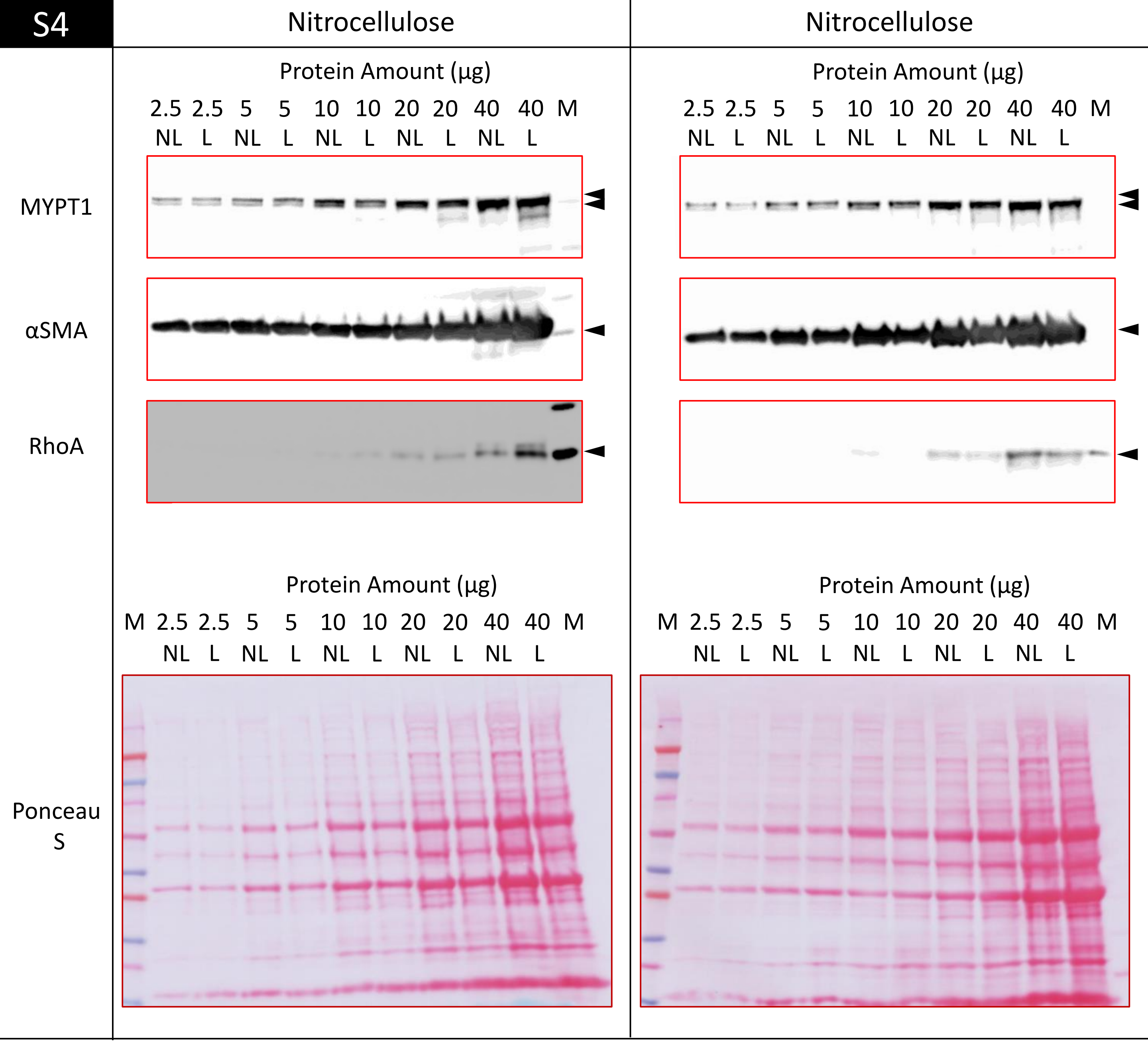
S4
Nitrocellulose
Nitrocellulose
Protein Amount (µg)
2.5 2.5 5 5 10 10 20 20 40 40 M
NL L NL L NL L NL L NL L
MYPT1
αSMA
RhoA
Protein Amount (µg)
M 2.5 2.5 5 5 10 10 20 20 40 40 M
NL L NL L NL L NL L NL L
Ponceau S

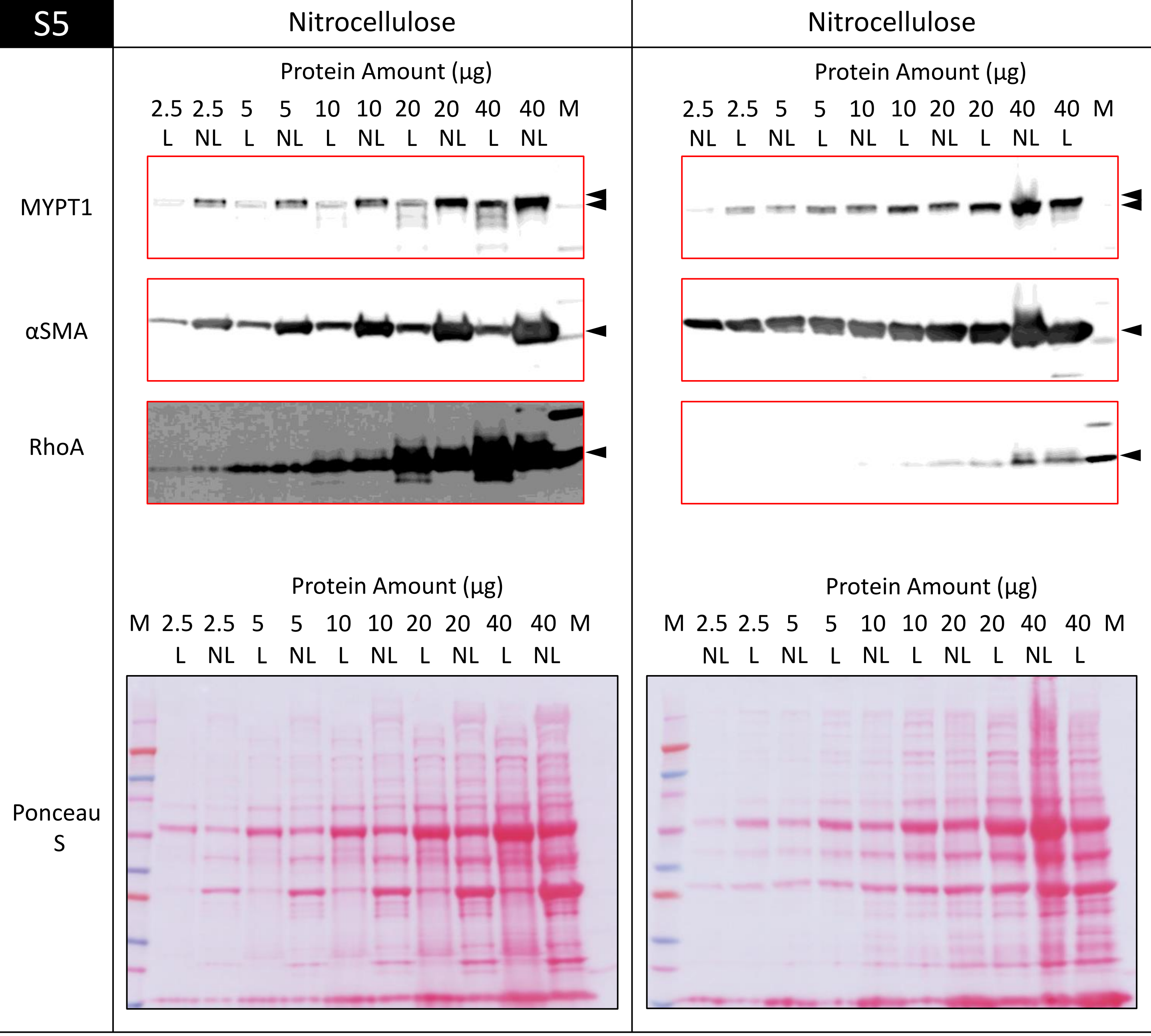
S5
Nitrocellulose
Nitrocellulose
Protein Amount (µg)
2.5 2.5 5 5 10 10 20 20 40 40 M
L NL L NL L NL L NL L NL
Protein Amount (µg)
2.5 2.5 5 5 10 10 20 20 40 40 M
NL L NL L NL L NL L NL L
MYPT1
αSMA
RhoA
Protein Amount (µg)
M 2.5 2.5 5 5 10 10 20 20 40 40 M
L NL L NL L NL L NL L NL
Protein Amount (µg)
M 2.5 2.5 5 5 10 10 20 20 40 40 M
NL L NL L NL L NL L NL L
Ponceau S

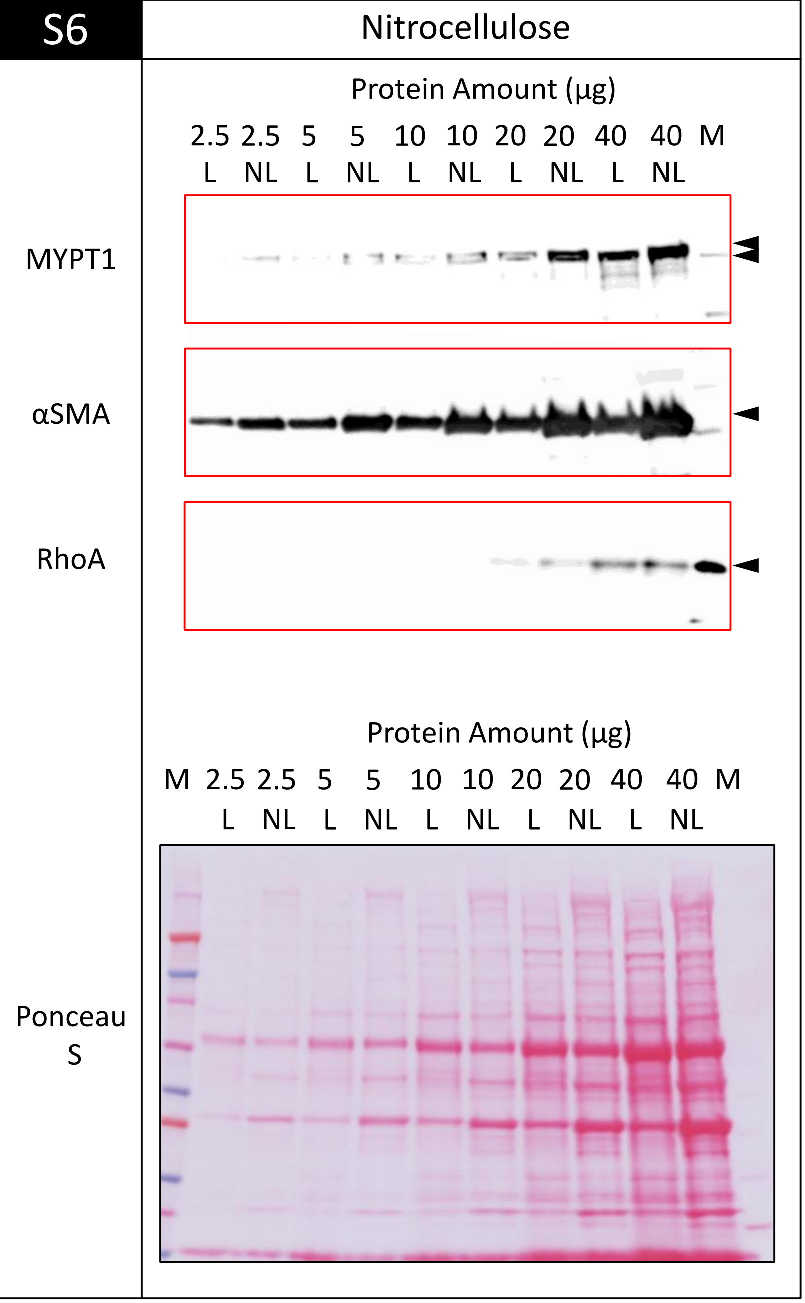
S6
Nitrocellulose
Protein Amount (µg)
2.5 2.5 5 5 10 10 20 20 40 40 M
L NL L NL L NL L NL L NL
MYPT1
αSMA
RhoA
Protein Amount (µg)
M 2.5 2.5 5 5 10 10 20 20 40 40 M
L NL L NL L NL L NL L NL
Ponceau S

Supplementary Figures S4, S5 and S6: Western blot Images from Results Figure 3. These are the Western blot images that were analysed in Figure 3. Each membrane contained a dilution series of myometrial lysate from 1 woman not-in-labour at term and 1 woman in-labor at term. Each membrane contained samples from different women. Samples were run at the indicated dilutions (noted as the amount of protein separated) and two molecular weight markers were used. Novex Sharp was used as a colorimetric marker (Left side of the Ponceau S Images) and MagicMark XP (Right side of the Ponceau S Images) was used for protein sizing following chemiluminescence detection.

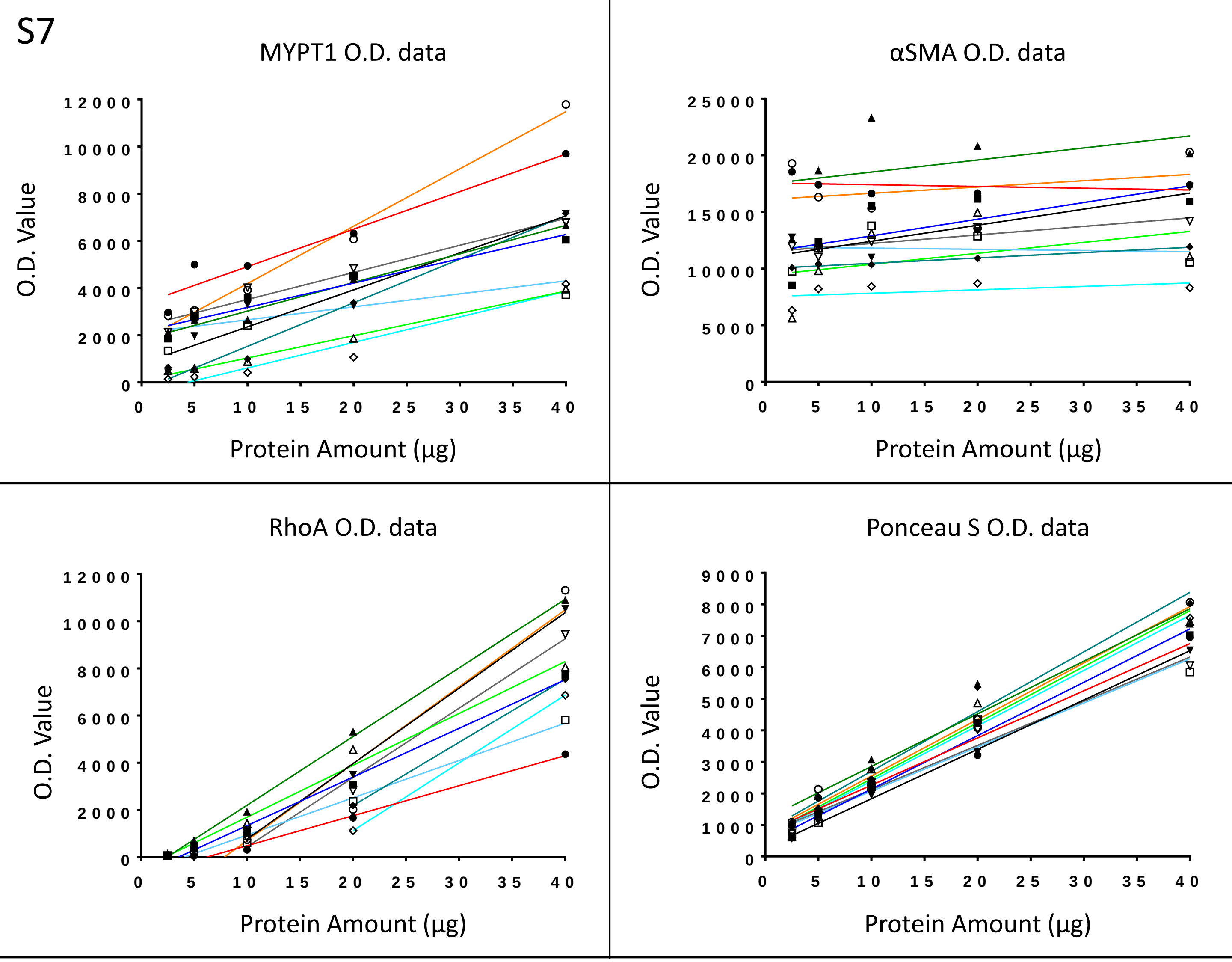

Supplementary Figure S7: Western blot O.D. Data from Results Figure 3. These are the Western blot O.D. data that were used for the normalisations in Results Figure 3. These data were obtained from the Western blots shown in supplementary figures S3, S4, and S5.

S8

A

Representative Ponceau S Stain of Blots used for Figure 4 (protein spiking)

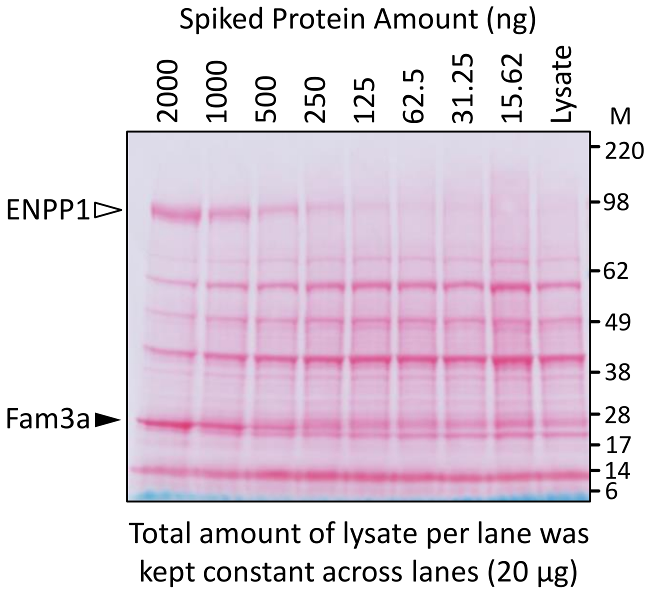

B

Ponceau S O. D. Values of Blots used in Figure 4
(entire lane)

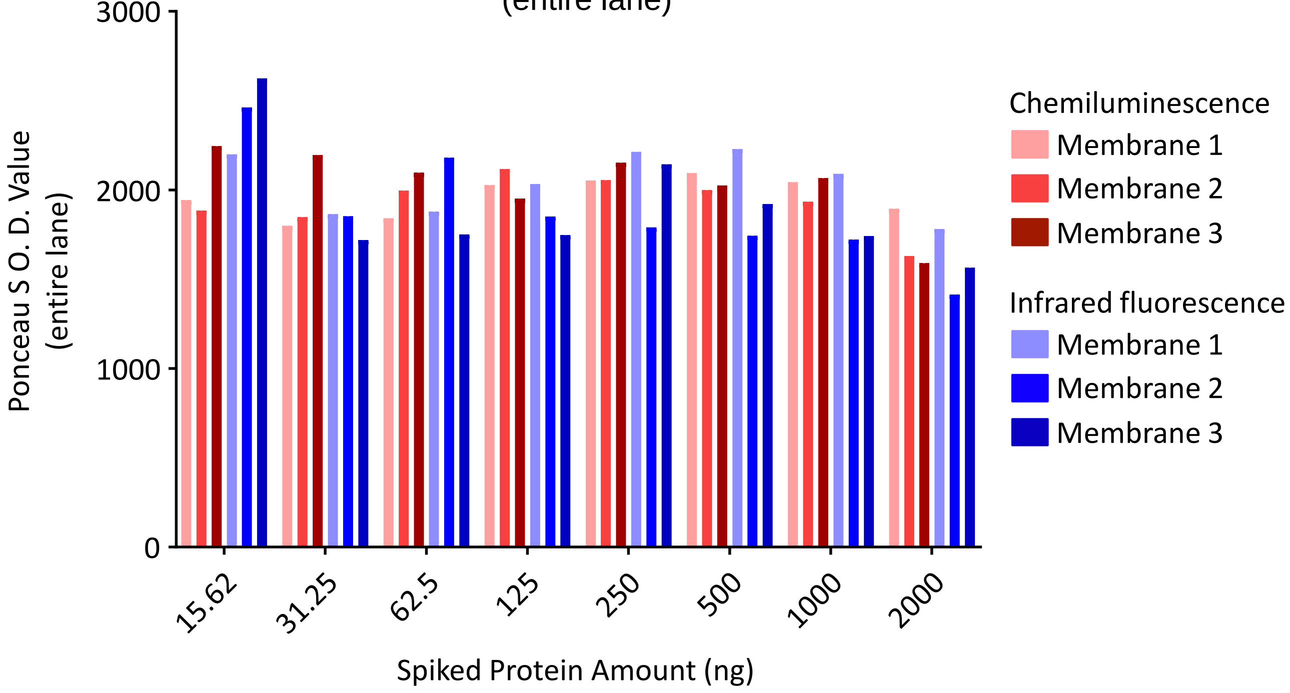

Supplementary Figure S8: Ponceau S Data for ENPP1 and Fam3a Protein Spiking Experiments. These membranes were the membranes used in Results Figure 4. A) Representative image of a Ponceau S stained membrane in this experiment. Each lane shows the lysates in which ENPP1 and Fam3a were 2-fold serially diluted while keeping total lysate amount similar between samples. The molecular weight marker was SeeBlue Plus 2 mixed 2:1 with MagicMark XP. The molecular weights listed here are from the colorimetric SeeBlue Plus 2 marker. B) O.D. data for each entire lane (listed by the amount of each spiked protein present) for all membranes used in this experiment.

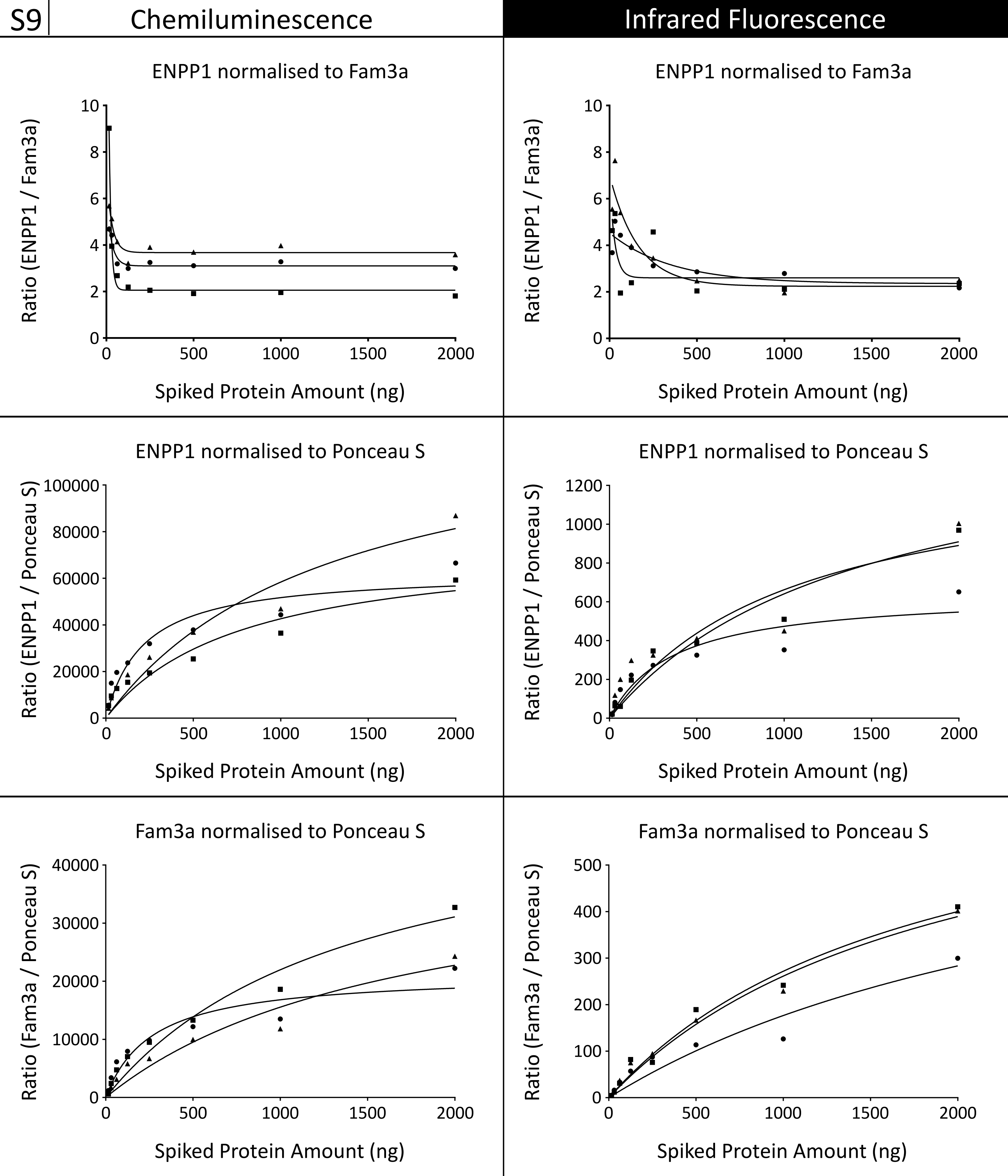
S9
Chemiluminescence
Infrared Fluorescence
ENPP1 normalised to Fam3a
Ratio (ENPP1 / Fam3a)
Spiked Protein Amount (ng)
ENPP1 normalised to Fam3a
Ratio (ENPP1 / Fam3a)
Spiked Protein Amount (ng)
ENPP1 normalised to Ponceau S
Ratio (ENPP1 / Ponceau S)
Spiked Protein Amount (ng)
ENPP1 normalised to Ponceau S
Ratio (ENPP1 / Ponceau S)
Spiked Protein Amount (ng)
Fam3a normalised to Ponceau S
Ratio (Fam3a / Ponceau S)
Spiked Protein Amount (ng)
Fam3a normalised to Ponceau S
Ratio (Fam3a / Ponceau S)
Spiked Protein Amount (ng)

Supplementary Figure S9: Normalised Data for ENPP1 and Fam3a Protein Spiking Experiments. These are the normalised data for the membranes used in Results Figure 4.
